# Supplementary material for: Three-dimensional ultrasound matrix imaging
Source: Nat Commun. 2023 Oct 25;14:6793. doi: 10.1038/s41467-023-42338-8 (PMC10600255; doi:10.1038/s41467-023-42338-8)
Supplement: Supplementary file 1 — Supplementary Information [file 41467_2023_42338_MOESM1_ESM.pdf]

Supplementary Information on Three-Dimensional Ultrasound Matrix Imaging

Flavien Bureau,[1] Justine Robin,[1,2] Arthur Le Ber,[1]
William Lambert,[1,3] Mathias Fink,[1] and Alexandre Aubry[1]

[1]*Institut Langevin, ESPCI Paris,*
PSL University, CNRS, 75005 Paris, France
[2]*Physics for Medecine, ESPCI Paris, PSL University,*
INSERM, CNRS, 75015 Paris, France
[3]*Hologic / SuperSonic Imagine,*
135 Rue Emilien Gautier, 13290 Aix-en-Provence, France

(Dated: September 27, 2023)

Abstract

This document provides further information on: (*i*) the UMI workflow; (*ii*) the RPSF and the common midpoint basis; (*iii*) the comparison between iterative time reversal and phase reversal; (*iv*) the bias of the $\mathbf{T}-$matrix estimator; (*v*) the comparison between a multi-scale and local analysis of wave distortions; (*vi*) the impact of the confocal filter; (*vii*) the effect of an incompleteness of the illumination basis.

I. WORKFLOW

Supplementary Figure 1 shows a workflow that sums up the different steps of the UMI procedure performed in the accompanying paper.

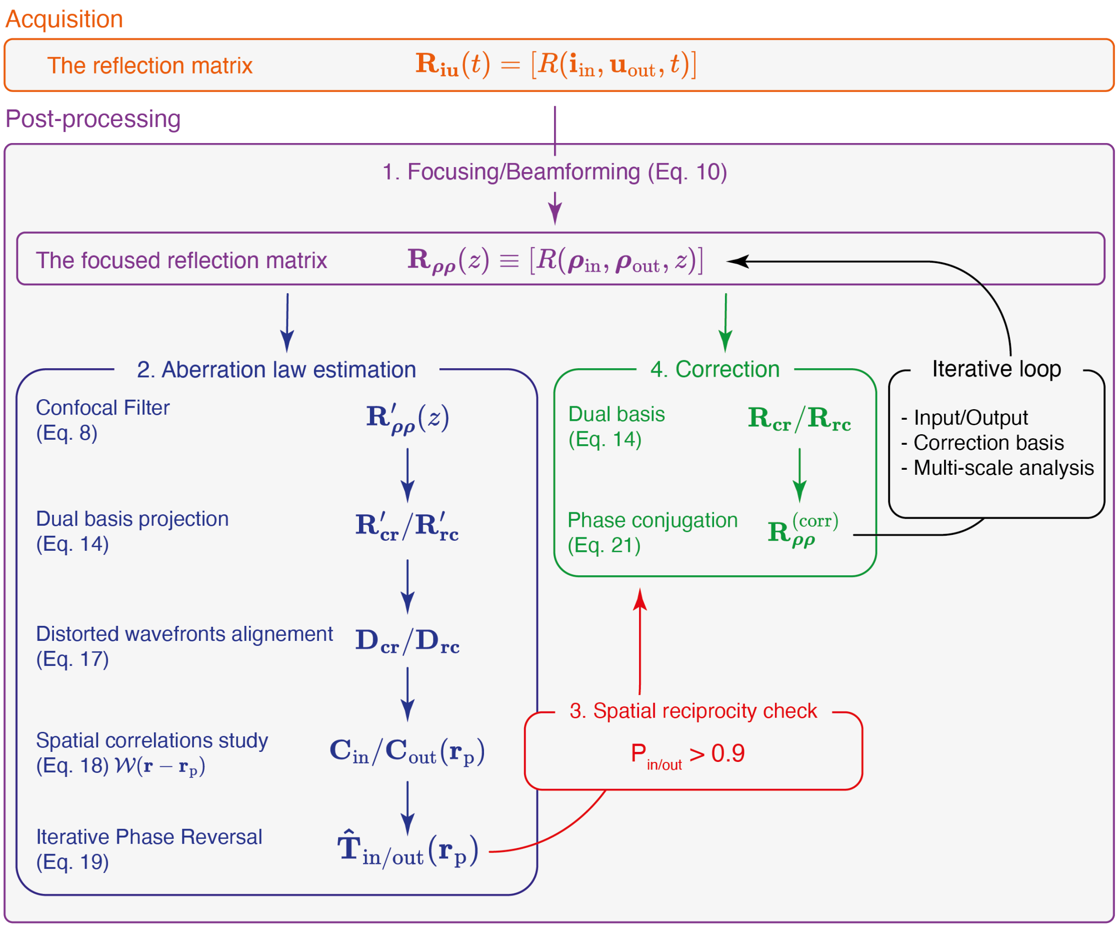

SUPPLEMENTARY FIGURE 1. **Flowchart of the UMI process.**

II. RPSF AND COMMON MIDPOINT

To probe the local focusing quality, the reflection point spread function (RPSF) can be investigated. Its extraction from the focused reflection matrix, $\mathbf{R}_{\rho\rho}(z) = [R(\boldsymbol{\rho}_{\mathrm{in}}, \boldsymbol{\rho}_{\mathrm{out}}, z)]$, consists in the following change of variable to project the data into

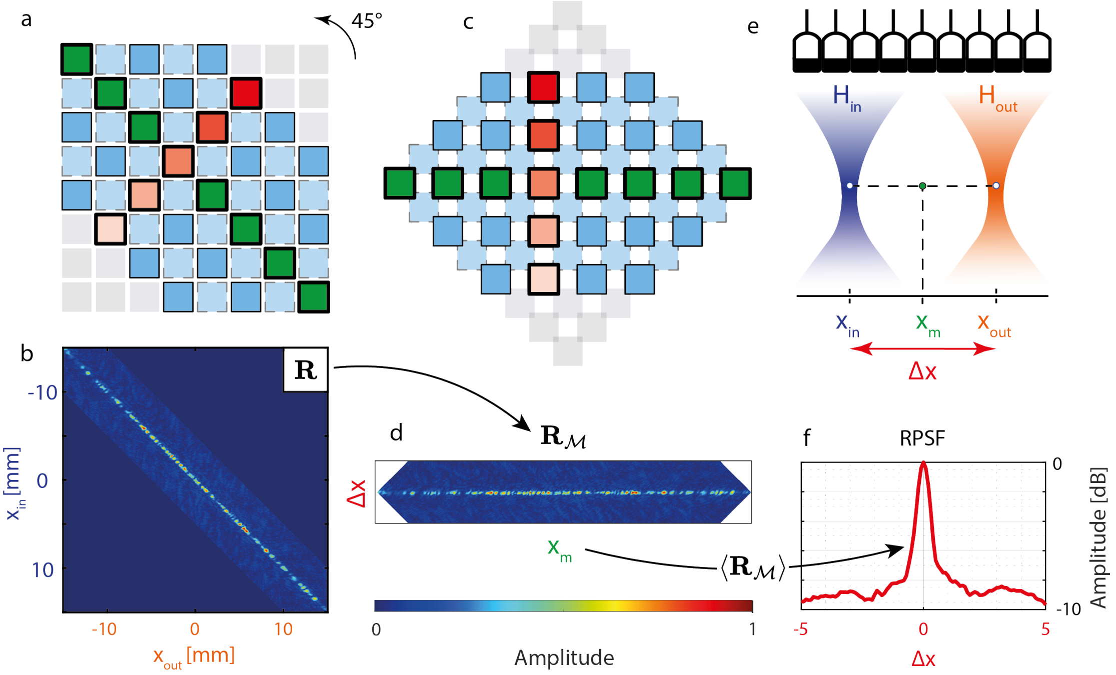

SUPPLEMENTARY FIGURE 2. **Common midpoint representation.** In 2D ultrasound imaging with linear or convex probes, the change from a (**a**,**b**) conventional to a (**c**,**d**) common midpoint representation corresponds to a 45° rotation of the focused reflection matrix. Panels a and c show a schematic representation of such a transformation, while panels b and d show experimental ultrasound data in speckle of an ultrasound phantom with a linear probe. Note that the change from the focused to the common midpoint representation implies two new sampling grids, represented by solid and dashed lines. (**e**) Schematic representation of the position of the input (x_{in}) and output (x_{out}) focal spots, spaced by Δx and their common midpoint x_{m}. (**f**) Extracted RPSF by spatial averaging over all midpoints x_{m} at depth $z = 30$ mm.

a common midpoint basis:

$$\underbrace{\begin{bmatrix} \boldsymbol{\rho}_{\mathrm{in}} \\ \boldsymbol{\rho}_{\mathrm{out}} \\ z \end{bmatrix}}_{\text{Focused}} \rightarrow \underbrace{\begin{bmatrix} \Delta\boldsymbol{\rho} \\ \boldsymbol{\rho}_m \\ z \end{bmatrix} = \begin{bmatrix} \boldsymbol{\rho}_{\mathrm{out}} - \boldsymbol{\rho}_{\mathrm{in}} \\ \frac{\boldsymbol{\rho}_{\mathrm{in}}+\boldsymbol{\rho}_{\mathrm{out}}}{2} \\ z \end{bmatrix}}_{\text{Common midpoint}}. \tag{1}$$

This operation is described schematically in Supplementary Figure 2 for the simple case of 2D imaging with a linear array of transducers. It consists in extracting each antidiagonal of the focused reflection matrix $\mathbf{R}_{xx}(z)$ (red boxes in Supplementary Figure 2a), corresponding to a matrix rotation by 45°. In this representation, $x_\mathrm{m} = (x_\mathrm{in} + x_\mathrm{out})/2$ is the common midpoint between the input and output focal spot, with the two separated by a distance $\Delta x = x_\mathrm{out} - x_\mathrm{in}$. These considerations can be extended to 3D imaging, so that the transverse coordinate, previously x, now becomes $\boldsymbol{\rho} = (x, y)$.

III. CORRELATION MATRIX OF WAVE DISTORTIONS

In the accompanying paper, an iterative phase reversal (IPR) process and a multi-scale analysis of $\mathbf{D}$ have been implemented to retrieve the $\mathbf{T}-$matrix. In the following, we provide a theoretical framework to justify this process, outline its limits and conditions of success. For sake of lighter notation, the dependence over $\mathbf{r}_\mathrm{p}$ will be omitted in the following.

At each step of the aberration correction process, a local correlation matrix of $\mathbf{D}$ is computed. The UMI process assumes the convergence of the correlation matrix $\mathbf{C}$ towards its ensemble average $\langle \mathbf{C} \rangle$, the so-called covariance matrix[1,2]. In fact, this convergence is never fully realized and $\mathbf{C}$ should be decomposed as the sum of this covariance matrix $\langle \mathbf{C} \rangle$ and a perturbation term $\delta\mathbf{C}$:

$$\mathbf{C} = \langle \mathbf{C} \rangle + \delta\mathbf{C}. \tag{2}$$

The intensity of the perturbation term scales as the inverse of the number $N_\mathcal{W} = (w_\rho^2 w_z)/(\delta\rho_0^2 \delta z_0)$ of resolution cells in each sub-region[1–3]:

$$\left\langle |\delta C(\mathbf{c}, \mathbf{c}', \mathbf{r}_\mathrm{p})|^2 \right\rangle = \frac{\left\langle |C(\mathbf{c}, \mathbf{c}', \mathbf{r}_\mathrm{p})|^2 \right\rangle}{N_\mathcal{W}} \tag{3}$$

This perturbation term can thus be reduced by increasing the size of the spatial window $\mathcal{W}$, but at the cost of a resolution loss. In the following, we express theoretically the bias induced by this perturbation term on the estimation of **T**-matrices. In particular, we will show how it scales with $N_{\mathcal{W}}$ in each spatial window $\mathcal{W}$ and the focusing quality. To that aim, we will consider the output correlation matrix $\mathbf{C}_{\text{out}}$ but a similar demonstration can be performed at input.

IV. COVARIANCE MATRIX: SYNTHESIS OF A VIRTUAL GUIDE STAR

Under assumptions of local isoplanicity in each spatial window and random reflectivity, the covariance matrix can be expressed as follows[1]:

$$\langle \mathbf{C}_{\text{out}} \rangle = \mathbf{T}_{\text{out}} \times \mathbf{C}_H \times \mathbf{T}_{\text{out}}^{\dagger}, \tag{4}$$

or in terms of matrix coefficients,

$$\langle \mathbf{C}(\mathbf{c}, \mathbf{c}') \rangle = T_{\text{out}}(\mathbf{c}) T^{*}_{\text{out}}(\mathbf{c}') \underbrace{\int d\boldsymbol{\rho} |H_{\text{in}}(\boldsymbol{\rho})|^2 e^{-i2\pi \frac{(\mathbf{c}-\mathbf{c}').\rho}{\lambda z_p}}}_{=C_H(\mathbf{c},\mathbf{c}')} . \tag{5}$$

$\mathbf{C}_H$ is a reference correlation matrix associated with a virtual reflector whose scattering distribution corresponds to the input focal spot intensity $|H_{\text{in}}(\boldsymbol{\rho})|^2$. This scatterer plays the role of virtual guide star in the UMI process (Fig. 1k of the accompanying paper).

V. COMPARISON BETWEEN ITERATIVE TIME REVERSAL AND PHASE REVERSAL

In previous works on 2D UMI[1,2], the $\mathbf{T}$-matrix was estimated by performing a singular value decomposition of $\mathbf{D_{rc}}$:

$$\mathbf{D_{rc}} = \mathbf{V}_{\text{in}}^{\dagger} \times \mathbf{\Sigma} \times \mathbf{U}_{\text{out}}, \tag{6}$$

or, equivalently, the eigenvalue decomposition of $\mathbf{C}_{\text{out}}$:

$$\mathbf{C}_{\text{out}} = \mathbf{U}_{\text{out}}^{\dagger} \times \mathbf{\Sigma}^2 \times \mathbf{U}_{\text{out}}. \tag{7}$$

$\mathbf{\Sigma}$ is a diagonal matrix containing the singular values σ_i in descending order: $\sigma_1 > \sigma_2 > .. > \sigma_N$. $\mathbf{U}_{\text{out}}$ and $\mathbf{V}_{\text{in}}$ are unitary matrices that contain the orthonormal set of output and input eigenvectors, $\mathbf{U}_{\text{out}}^{(i)} = [U_{\text{out}}^{(i)}(\mathbf{c})]$ and $\mathbf{V}_{\text{in}}^{(i)} = [V_{\text{in}}^{(i)}(\mathbf{r})]$.

The reason of this eigenvalue decomposition can be intuitively understood by considering the asymptotic case of a point-like input focusing beam. In this ideal case, Eq. 7 becomes $C_{\text{out}}(\mathbf{c}, \mathbf{c}') = T_{\text{out}}(\mathbf{c})T^*_{\text{out}}(\mathbf{c}')$. $\mathbf{D_{rc}}$ is then of rank 1 – the first output singular vector $\mathbf{U}_{\text{out}}^{(1)}$ yields the aberration transmittance $\mathbf{T}_{\text{out}}$.

However, in reality, the input PSF H_{in} is of course far from being point-like. The spectrum of $\mathbf{D_{rc}}$ displays a continuum of singular values [Supplementary Figure 3d]. The effective rank of $\mathbf{C}_{\text{out}}$ is shown to scale as the number of resolution cells covered by the input PSF H_{in}[2]:

$$M_\delta \sim (\delta\rho_{\text{in}}/\delta\rho_0)^2. \tag{8}$$

with $\delta\rho_{\text{in}}$ the spatial extension of H_{in}. The amplitude of the corresponding eigenvectors $\mathbf{U}_{\text{out}}^{(i)}$ depends on the exact shape of the virtual guide star, that is to say, on aberrations induced by the incident wave-front.

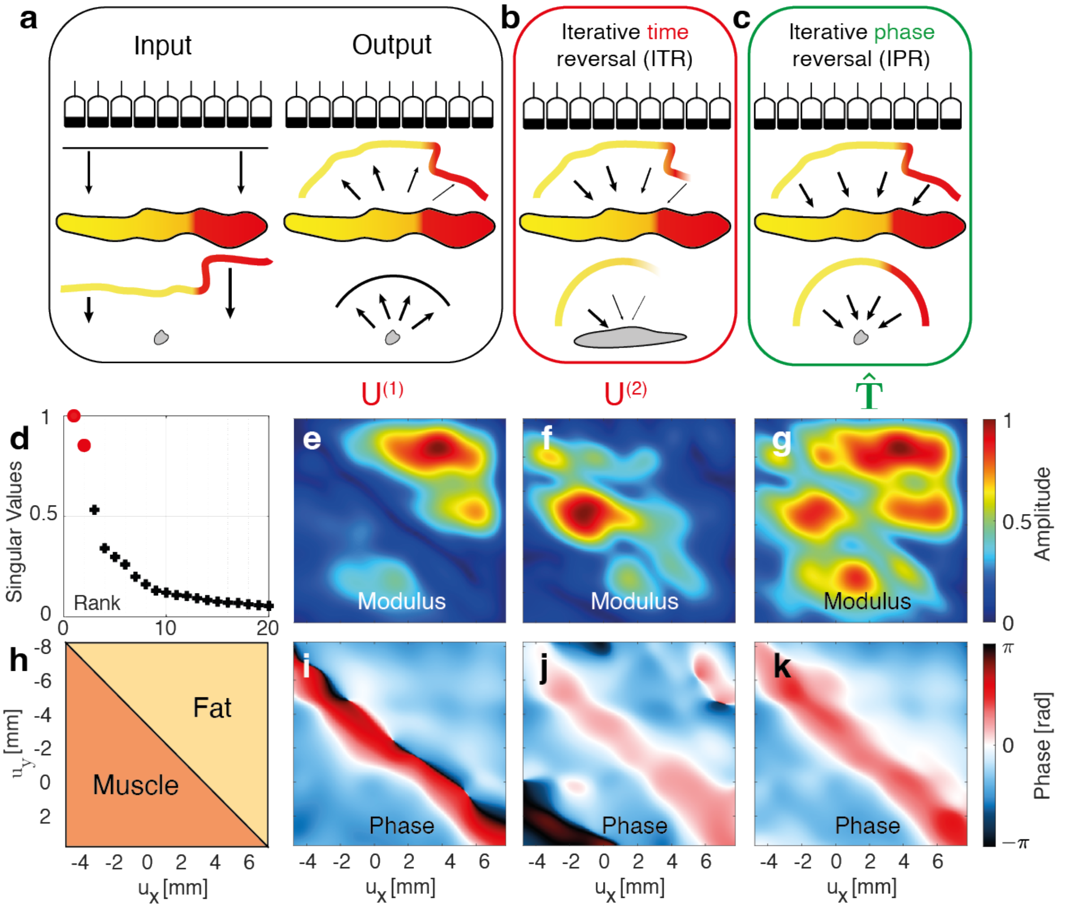

SUPPLEMENTARY FIGURE 3. **Iterative Time Reversal vs. Iterative Phase Reversal.** (**a**) The first step of ITR and IPR corresponds to the following fictitious experiment: Insonifying the medium by an arbitrary wave-front (here a plane wave) using an array of transducers and recording the reflected wave-front with the same probe. (**b**) The ITR process consists in time-reversing this wave-front in post-processing and sending it back into the medium, recording again the reflected wave-front, and so on. (**c**) The IPR process is similar but normalizes the amplitude of the time-reversed wavefront at every iteration. (**d**) Singular value distribution of $\mathbf{D_{rc}}$ for a box $\mathcal{W}$ of dimension $\mathbf{w} = (w_x, w_y, w_z) = (2, -5, 2)$mm centered around point $\mathbf{r_p} = (3, -5.5, 23)$ mm. (**e**,**f**) Modulus of the two first eigenvectors $\mathbf{U}_{\text{out}}^{(i)}$. (**g**) Modulus of the vector $\mathbf{C}_{\text{out}} \times \hat{\mathbf{T}}_{\text{out}}$. (**h**) Delimitation of muscle and fat over the probe surface. (**i**,**j**,**k**) Phase of $\mathbf{U}_{\text{out}}^{(1)}$, $\mathbf{U}_{\text{out}}^{(2)}$ and $\hat{\mathbf{T}}_{\text{out}}$.

Supplementary Figures 3e and f show the modulus of two first eigenvectors, $\mathbf{U}_{\text{out}}^{(1)}$

and $\mathbf{U}_{\text{out}}^{(2)}$. They clearly show a complementary feature. While $\mathbf{U}_{\text{out}}^{(1)}$ is associated with the fat layer, $\mathbf{U}_{\text{out}}^{(2)}$ maps onto the muscle part of the pork chop [Supplementary Figure 3h]. This result can be understood by the discontinuity of the speed-of-sound between the muscle and fat parts of the pork chop that breaks the spatial invariance and isoplanicity. As a consequence, the SVD process tends to converge onto eigenstates associated with the most isoplanatic components of $\mathbf{D}_{\mathbf{rc}}$.

This property is not satisfactory in the present case since each eigenvector only covers a part of the probe aperture. In other words, the phases of $\mathbf{U}^{(1)}$ [Supplementary Figure 3i] and $\mathbf{U}^{(2)}$ [Supplementary Figure 3j] are only satisfying estimators of $\mathbf{T}$ over some parts of the probe. Therefore, they cannot independently lead to an aberration compensation over the full numerical aperture.

To circumvent that problem, one can take advantage of the analogy with iterative time reversal (ITR). The eigenvector $\mathbf{U}_{\text{out}}^{(1)}$ can actually be seen as the result of the following fictitious experiment that consists in illuminating the virtual scatterer by an arbitrary wave-front and recording the reflected wave-field [Supplementary Figure 3a]. This wave-field is time-reversed and back-emitted towards the virtual scatterer [Supplementary Figure 3b]. This process can then be iterated many times and each step can be mathematically written as:

$$\sigma \mathbf{W}^{(n+1)} = \mathbf{C}_{\text{out}} \times \mathbf{W}^{(n)} \tag{9}$$

with $\mathbf{W}^{(n)}$, the wave-front at iteration n of the ITR process and σ, the scatterer reflectivity. ITR is shown to converge towards a time-reversal invariant that is nothing other than the first eigenvector, $\mathbf{U}_{\text{out}}^{(1)} = \lim_{n \to +\infty} \mathbf{W}^{(n)}$.

To optimize the estimation of aberrations over the full probe aperture, our idea is to modify the ITR process by still re-emitting a phase-reversed wave-field but with a constant amplitude on each probe element [Supplementary Figure 3c]. In

practice, this operation is performed using the following IPR algorithm:

$$\hat{\mathbf{T}}_{\text{out}}^{(n+1)} = \exp\left[i \arg\left\{\mathbf{C}_{\text{out}} \times \hat{\mathbf{T}}_{\text{out}}^{(n)}\right\}\right] \tag{10}$$

where $\hat{\mathbf{T}}_{\text{out}}^{(n)}$ is the estimator of $\mathbf{T}_{\text{out}}$ at the nth iteration of IPR. $\hat{\mathbf{T}}_{\text{out}}^{(0)}$ is an arbitrary wave-front that initiates IPR (typically a plane wave). $\hat{\mathbf{T}}_{\text{out}} = \lim_{n\to\infty} \hat{\mathbf{T}}_{\text{out}}^{(n)}$ is the result of this IPR process. Unlike ITR, IPR equally addresses each angular component of the imaging process to reach a diffraction-limited resolution. Supplementary Figure 3g illustrates this fact by showing the modulus of $\mathbf{C}_{\text{out}} \times \hat{\mathbf{T}}_{\text{out}}$. Compared with $\mathbf{U}_{\text{out}}^{(1)}$ [Supplementary Figure 3e] and $\mathbf{U}_{\text{out}}^{(2)}$ [Supplementary Figure 3f], it clearly shows that the phase-reversed invariant $\hat{\mathbf{T}}_{\text{out}}$ simultaneously addresses each angular component of the aberrated wave-field. $\hat{\mathbf{T}}_{\text{out}}$ is thus a much better estimator of the $\mathbf{T}-$matrix [Supplementary Figure 3k] than the aberration phase laws extracted by the SVD process [Supplementary Figures 3i and j].

When applied to the whole field-of-view, the IPR algorithm is mathematically equivalent to the CLASS algorithm developed in optical microscopy[4]. However, the IPR algorithm is much more efficient for a local compensation of aberrations. For IPR, the angular resolution $\delta\theta$ of the aberration phase law is only limited by the angular pitch of the plane wave illumination basis or the pitch p of the transducer array in the canonical basis: $\delta\theta_I \sim \lambda/p$. With CLASS, the resolution $\delta\theta_C$ of the aberration law is governed by the size of the spatial window $\mathcal{W}$ on which the focused reflection matrix is truncated: $\delta\theta_C \sim z/w_\rho$. It can be particularly detrimental when high-order aberrations and small isopalanatic patches are targeted.

VI. BIAS ON THE T−MATRIX ESTIMATION

In practice, however, the $\mathbf{T}-$matrix estimator is still impacted by the blurring of the synthesized guide star and the presence of diffusive background and/or noise. Therefore, the whole process shall be iterated at input and output in order

to gradually refine the guide star and reduce the bias on our $\mathbf{T}-$matrix estimator. Moreover, the spatial window $\mathcal{W}$ over which the $\mathbf{C}-$matrix is computed shall be gradually decreased in order to address the high-order aberration components, the latter one being associated with smaller isoplanatic patches.

To understand the parameters controlling the bias $\delta\mathbf{T}_{\text{out}}$ between $\hat{\mathbf{T}}_{\text{out}}$ and $\mathbf{T}_{\text{out}}$, one can express $\hat{\mathbf{T}}_{\text{out}}$ as follows:

$$\hat{\mathbf{T}}_{\text{out}} = \exp\left(j\arg\left\{\mathbf{C}_{\text{out}} \times \hat{\mathbf{T}}_{\text{out}}\right\}\right) = \frac{\mathbf{C}_{\text{out}} \times \hat{\mathbf{T}}_{\text{out}}}{||\mathbf{C}_{\text{out}} \times \hat{\mathbf{T}}_{\text{out}}||} \quad (11)$$

By injecting Eq. 2 into the last expression, $\hat{\mathbf{T}}_{\text{out}}$ can be expressed, at first order, as the sum of its expected value $\mathbf{T}_{\text{out}}$ and a perturbation term $\delta\hat{\mathbf{T}}_{\text{out}}$:

$$\hat{\mathbf{T}}_{\text{out}} = \underbrace{\frac{\langle\mathbf{C}_{\text{out}}\rangle \times \mathbf{T}_{\text{out}}}{||\langle\mathbf{C}_{\text{out}}\rangle \times \mathbf{T}_{\text{out}}||}}_{=\mathbf{T}_{\text{out}}} + \underbrace{\frac{\delta\mathbf{C}_{\text{out}} \times \mathbf{T}_{\text{out}}}{||\langle\mathbf{C}_{\text{out}}\rangle \times \mathbf{T}_{\text{out}}||}}_{\simeq\delta\hat{\mathbf{T}}_{\text{out}}}. \quad (12)$$

The bias intensity can be expressed as follows:

$$|\delta\mathbf{T}_{\text{out}}|^2 = \frac{\mathbf{T}_{\text{out}}^\dagger \times \delta\mathbf{C}_{\text{out}}^\dagger \times \delta\mathbf{C}_{\text{out}} \times \mathbf{T}_{\text{out}}}{\mathbf{T}_{\text{out}}^\dagger \times \langle\mathbf{C}_{\text{out}}\rangle^\dagger \times \langle\mathbf{C}_{\text{out}}\rangle \times \mathbf{T}_{\text{out}}} \quad (13)$$

Using Eq. 3, the numerator of the last equation can be expressed as follows:

$$\mathbf{T}_{\text{out}}^\dagger \times \delta\mathbf{C}_{\text{out}}^\dagger \times \delta\mathbf{C}_{\text{out}} \times \mathbf{T}_{\text{out}} = N_u^2\langle|\delta C(\mathbf{c},\mathbf{c}')|^2\rangle = N_u^2|C(\mathbf{c},\mathbf{c})|^2/N_{\mathcal{W}}. \quad (14)$$

with N_u the number of transducers.

The denominator of Eq. 13 can be expressed as follows:

$$\mathbf{T}_{\text{out}}^\dagger \times \langle\mathbf{C}_{\text{out}}\rangle^\dagger \times \langle\mathbf{C}_{\text{out}}\rangle \times \mathbf{T}_{\text{out}} = M^2\left|\sum_{\mathbf{c}} T_{\text{in}} \overset{\mathbf{c}}{\circledast} T_{\text{in}}(\mathbf{c})\right|^2 \quad (15)$$

The bias intensity is thus given by:

$$|\delta T_{\text{out}}(\mathbf{c})|^2 = \frac{\left| T_{\text{in}} \overset{\mathbf{c}}{\circledast} T_{\text{in}}(\mathbf{0}) \right|^2}{N_{\mathcal{W}} \left| \sum_{\mathbf{c}} T_{\text{in}} \overset{\mathbf{c}}{\circledast} T_{\text{in}}(\mathbf{c}) \right|^2} \tag{16}$$

In the last expression, we recognize the ratio between the coherent intensity (energy deposited exactly at focus) and the mean incoherent input intensity. This quantity is known as the coherence factor in ultrasound imaging[3,5]:

$$\mathcal{C}_{\text{in}} = \frac{\sum_{\mathbf{c}} T_{\text{in}} \overset{\mathbf{c}}{\circledast} T_{\text{in}}(\mathbf{c})}{T_{\text{in}} \overset{\mathbf{c}}{\circledast} T_{\text{in}}(\mathbf{0})} = \frac{|H_{\text{in}}(\boldsymbol{\rho} = \mathbf{0})|^2}{\Delta\rho_{\max}^{-2} \int d\boldsymbol{\rho} |H_{\text{in}}(\boldsymbol{\rho})|^2} \tag{17}$$

In the speckle regime and for a 2D probe, the coherence factor $\mathcal{C}$ ranges from 0, for strong aberrations and/or multiple scattering background, to 4/9 in the ideal case[6]. The bias intensity can thus be rewritten as:

$$|\delta T_{\text{out}}(\mathbf{c})|^2 = \frac{1}{\mathcal{C}_{\text{in}}^2 N_{\mathcal{W}}} \tag{18}$$

This last expression justifies the multi-scale analysis proposed in the accompanying paper. A gradual increase of the focusing quality, quantified by $\mathcal{C}$, is required to address smaller spatial windows that scale as $N_{\mathcal{W}}$. Following this scheme, the bias made of our $\mathbf{T}-$matrix estimator can be minimized.

VII. PROBING THE BIAS INTENSITY WITH SPATIAL RECIPROCITY

In the accompanying paper, we use the scalar product $P_{\text{in/out}}$ between input and output aberration phase laws to monitor the bias $|\delta T|^2$ of our $\mathbf{T}-$matrix estimator. Here we demonstrate the link between both quantities. To do so, the estimator

can be written as:

$$\hat{T}(\mathbf{c}, \mathbf{r}_\mathrm{p}) = \exp\left[j\left\{\phi(\mathbf{c}, \mathbf{r}_\mathrm{p}) + \delta\phi(\mathbf{c}, \mathbf{r}_\mathrm{p})\right\}\right] \tag{19}$$

with $T(\mathbf{c}, \mathbf{r}_\mathrm{p}) = \exp\left[j\phi(\mathbf{c}, \mathbf{r}_\mathrm{p})\right]$ and $\delta\phi(\mathbf{c}, \mathbf{r}_\mathrm{p})$ the phase error of the estimator.

On the one hand, the bias intensity can be rewritten using Eq. 19 as follows:

$$|\delta T(\mathbf{c}, \mathbf{r}_\mathrm{p})|^2 = |1 - \exp[j\delta\phi(\mathbf{c}, \mathbf{r}_\mathrm{p})]|^2 = 4\sin^2\left[\frac{\delta\phi(\mathbf{c}, \mathbf{r}_\mathrm{p}))}{2}\right] \overset{\delta\phi<<1}{\sim} [\delta\phi(\mathbf{c}, \mathbf{r}_\mathrm{p})]^2 \tag{20}$$

On the other hand, the scalar product $P_{\mathrm{in/out}}$ is given by

$$P_{\mathrm{in/out}} = N_c^{-1} \sum_{\mathbf{c}} \exp\left[j\left\{\delta\phi_{\mathrm{in}}(\mathbf{c}, \mathbf{r}_\mathrm{p}) - \delta\phi_{\mathrm{out}}(\mathbf{c}, \mathbf{r}_\mathrm{p})\right\}\right] \tag{21}$$

In the previous equation, the sum over $\mathbf{c}$ can be replaced by an ensemble average since $N_c = N_u >> 1$:

$$P_{\mathrm{in/out}} = \left\langle \exp\left[j\left\{\delta\phi_{\mathrm{in}}(\mathbf{c}, \mathbf{r}_\mathrm{p}) - \delta\phi_{\mathrm{out}}(\mathbf{c}, \mathbf{r}_\mathrm{p})\right\}\right]\right\rangle . \tag{22}$$

Assuming a small phase error ($\delta\phi_{\mathrm{in/out}} << 1$), the last equation can be rewritten as follows

$$P_{\mathrm{in/out}} \simeq 1 + j\left\langle \delta\phi_{\mathrm{in}}(\mathbf{c}, \mathbf{r}_\mathrm{p}) - \delta\phi_{\mathrm{out}}(\mathbf{c}, \mathbf{r}_\mathrm{p})\right\rangle - \frac{\langle[\delta\phi_{\mathrm{in}}(\mathbf{c}, \mathbf{r}_\mathrm{p}) - \delta\phi_{\mathrm{out}}(\mathbf{c}, \mathbf{r}_\mathrm{p})]^2\rangle}{2}. \tag{23}$$

Since $\langle\delta\phi_{\mathrm{in/out}}\rangle = 0$ and $\langle\delta\phi_{\mathrm{in}}\delta\phi_{\mathrm{out}}\rangle = 0$, the last expression simplifies into

$$P_{\mathrm{in/out}} \simeq 1 - \frac{\langle|\delta\phi_{\mathrm{in}}(\mathbf{c}, \mathbf{r}_\mathrm{p})|^2}{2} - \frac{\langle|\delta\phi_{\mathrm{out}}(\mathbf{c}, \mathbf{r}_\mathrm{p})|^2\rangle}{2}. \tag{24}$$

Assuming an equivalent phase error at input and output ($\langle|\delta\phi_{\mathrm{in}}(\mathbf{c}, \mathbf{r}_\mathrm{p})|^2\rangle =$

$\langle|\delta\phi_{\text{out}}(\mathbf{c},\mathbf{r}_{\text{p}})|^2\rangle)$ finally leads to:

$$P_{\text{in/out}} \simeq 1 - \langle|\delta\phi(\mathbf{c},\mathbf{r}_{\text{p}})|^2\rangle. \tag{25}$$

Combining the latter expression with Eq. 20 leads to the final result:

$$P_{\text{in/out}} \simeq 1 - \left\langle|\delta T(\mathbf{c},\mathbf{r}_{\text{p}})|^2\right\rangle. \tag{26}$$

$P_{\text{in/out}}$ is thus a relevant quantity to estimate the bias intensity (see Fig. 3b of the accompanying paper).

VIII. MULTI-SCALE ANALYSIS OF WAVE DISTORTIONS

Supplementary Figure 4 demonstrates the benefit of a multi-scale analysis of wave distortions with a gradual decrease of spatial windows $\mathcal{W}$ at each step of the UMI process [Supplementary Figure 4a]. To that aim, this aberration correction scheme is compared with a direct estimation of the $\mathbf{T}-$matrix over the smallest patches $\mathcal{W}$ [Supplementary Figure 4d]. The estimated transmission matrices $\hat{\mathbf{T}}$ differ in both cases (see comparison between Supplementary Figures 4b and e) especially in the fat layer. The RPSFs obtained after phase conjugation of $\hat{\mathbf{T}}$ demonstrate the benefit of the multi-scale analysis [Supplementary Figure 4c] compared with a direct local investigation of wave distortions [Supplementary Figure 4f]. The fat area is actually the most aberrated in the field-of-view (see initial RPSFs displayed by Fig. 2b of the accompanying paper). The initial coherence factor $\mathcal{C}$ is thus much smaller in this area, which induces a strong bias on $\mathbf{T}$ when wave distortions are investigated over a reduced isoplanatic patch. On the contrary, a multi-scale analysis enables a gradual enhancement of this coherence factor in this area and finally leads to an unbiased estimation of $\mathbf{T}$.

Supplementary Figure 5 shows the performance of UMI by comparing the

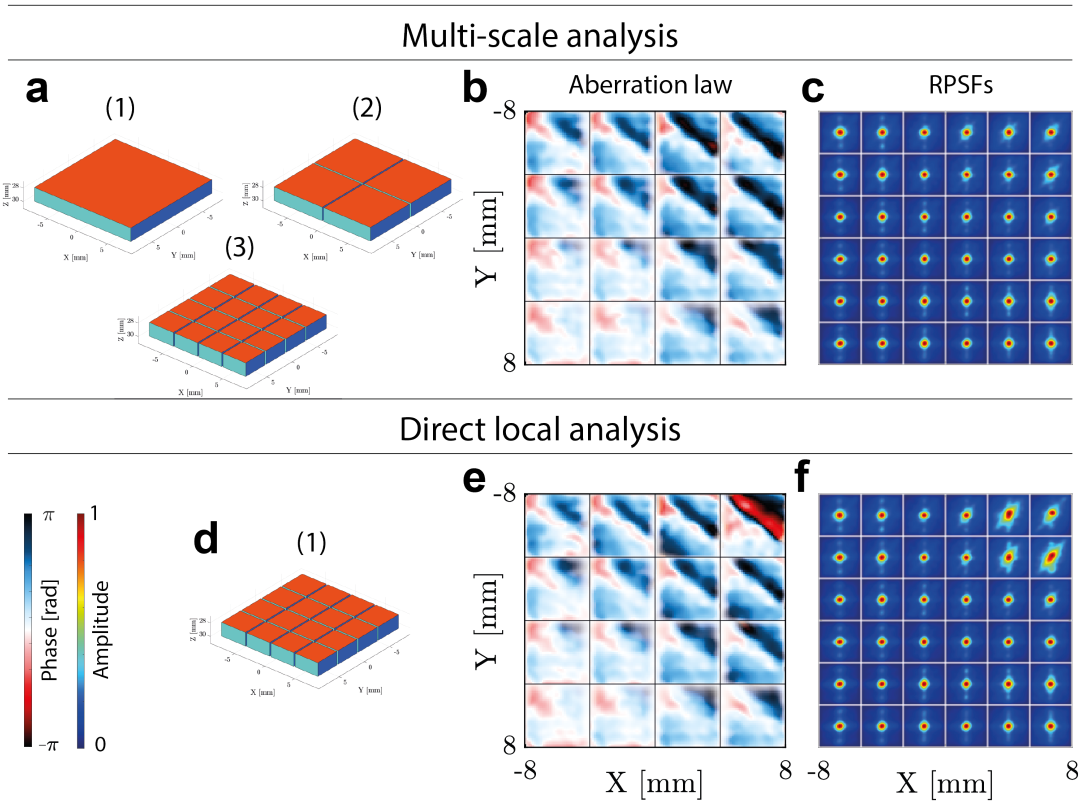

SUPPLEMENTARY FIGURE 4. **Multi-scale *versus* direct local analysis of wave distortions** (pork chop experiment, $z = 29$ mm). (**a**) Representation of the spatial windows used at each step of UMI (see Tab. III of the accompanying paper). (**b**) Aberration phase laws ($\hat{\mathbf{T}}$) extracted by a multi-scale analysis. (**c**) RPSFs after multi-scale aberration compensation. (**d**) Representation of the spatial windows used for a direct local compensation of wave distortions. (**e**) Aberration phase laws ($\hat{\mathbf{T}}$) extracted by a local analysis of **D**. (**f**) RPSFs after local aberration compensation.

RPSFs before and after aberration compensation. In the most aberrated area (top right of the field-of-view), the resolution is improved by almost a factor two, while the contrast is increased by 4.2 dB.

Supplementary Figure 6 shows the evolution of the RPSF during the UMI process applied to the head phantom experiment. A gradual enhancement of the focusing process is observed at each step of UMI, which enables an estimation of the $\mathbf{T}-$matrix at a higher resolution.

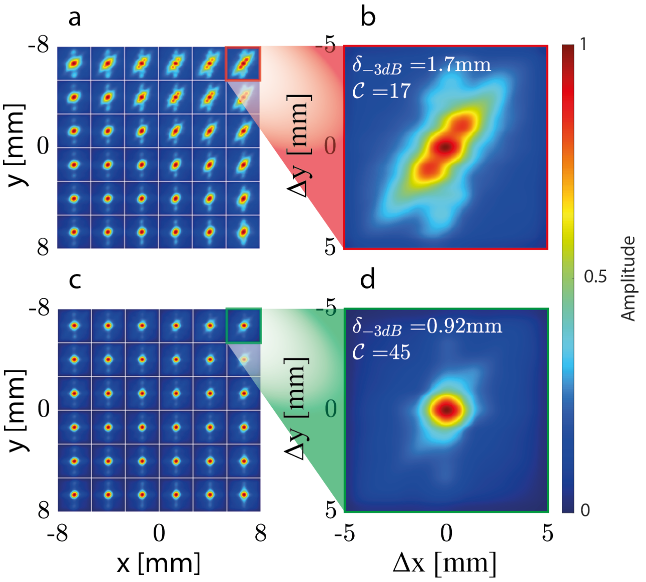

SUPPLEMENTARY FIGURE 5. **Contrast & resolution enhancement in the pork chop experiment.** (**a**) Maps of local RPSF ($z = 29$ mm). (**b**) Local RPSF on the top right of the field-of-view. (**c**) Map of RPSF after the UMI process. (**d**) Corrected RPSF on the top right of the field-of-view. The resolution is evaluated at -3dB (see Methods in the accompanying paper). The contrast $\mathcal{F}$ is the ratio between the confocal peak and the multiple scattering/noise background (see also Methods).

IX. CONFOCAL FILTER

Supplementary Figure 7 shows the effect of the confocal filter on the $\mathbf{T}-$matrix estimation. The output aberration phase laws contained in $\hat{\mathbf{T}}_{\text{out}}$ look much more noisy in absence of an adaptive confocal filter (see the comparison between Supplementary Figures 7a and b). As shown by the scalar product between input and output aberration phase laws [Supplementary Figure 7c], this "noise" comes from the imperfect convergence of $\hat{\mathbf{T}}$ towards $\mathbf{T}$. Without any confocal filter, multiple scattering drastically reduces the coherence factor and induces a strong bias on estimation of $\mathbf{T}$ (see Supplementary Section S5). On the contrary, the adaptive confocal filter enables an enhancement of this coherence factor $\mathcal{C}$ to ensure a satisfactory estimation of $\mathbf{T}$. The high degree of correlation between $\hat{\mathbf{T}}_{\text{in}}$

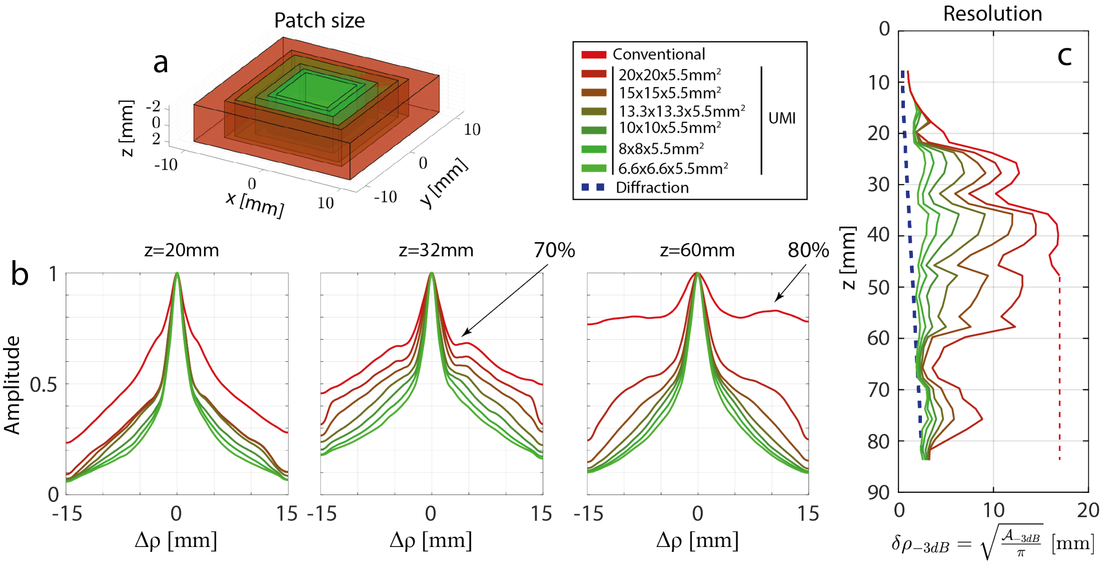

SUPPLEMENTARY FIGURE 6. **Multi-scale compensation of wave distortions in the head phantom.** (**a**) Successive patches used to perform a multi-scale analysis of wave distortions. (**b**) Radial profile of the RPSF amplitude at each step for three different depths (From left to right: $z = 20$, $z = 32$ and $z = 60$ mm). (**c**) Resolution as a function of depth at each step of correction (from red to green). At large depth (red dashed line), initial resolution can not be extracted as the incoherent background is larger than 1/2 as shown in panel (**b**) for $z = 60$ mm.

and $\hat{\mathbf{T}}_{\text{out}}$ proves this last assertion [Supplementary Figure 7d]. The effect of the confocal filter is also particularly obvious when looking at the RPSF obtained at the end of the UMI process. While a strong incoherent background subsists on the lateral parts of the field-of-view when no confocal filter is applied [Supplementary Figure 7e], a homogeneous focusing quality is obtained with the confocal filter [Supplementary Figure 7f].

X. ILLUMINATION BASIS

Supplementary Figure 8 shows the impact of the illumination sequence on UMI. If the input illumination basis is complete [Supplementary Figure 8a], the RSPF exhibits the expected diffraction-limited resolution [Supplementary Figure 8f]. The

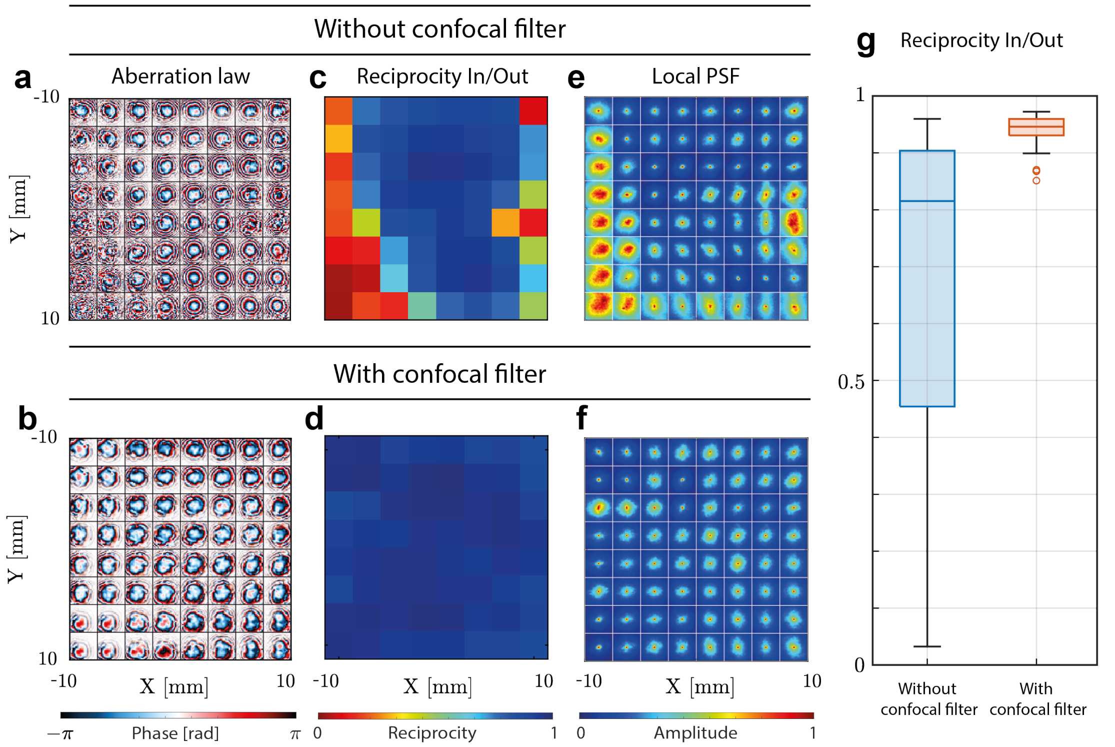

SUPPLEMENTARY FIGURE 7. **Confocal filter in transcranial imaging.** (**a**,**b**) Output aberration phase laws ($\hat{\mathbf{T}}_{\text{out}}$) extracted without and with a confocal filter. (**c**,**d**) Normalised scalar products $P_{\text{in/out}}$ without and with a confocal filter, respectively. (**e**,**f**) RPSFs obtained with UMI without and with a confocal filter. (**g**) Box plot corresponding to the panels (**c**,**d**). Experimental data shown in this figure correspond to the head phantom experiment described in the accompanying paper ($z = 50mm$).

side lobes along the y-axis are due to the probe geometry made of four blocks of transducers separated by a distance of 0.5 mm (three inactive rows of transducers along the y-axis).

When the number of illuminating plane waves is reduced [Supplementary Figures 8b-e], spatial aliasing occurs on corresponding RPSFs [Supplementary Figures 8g-j]. The maximal extension $\Delta\rho_{\text{max}}$ of the RPSF has to be fixed to avoid the spatial aliasing induced by the incompleteness of the plane wave illumination basis; $\Delta\rho_{\text{max}}$ is inversely proportional to the angular step $\delta\theta$ of the plane wave

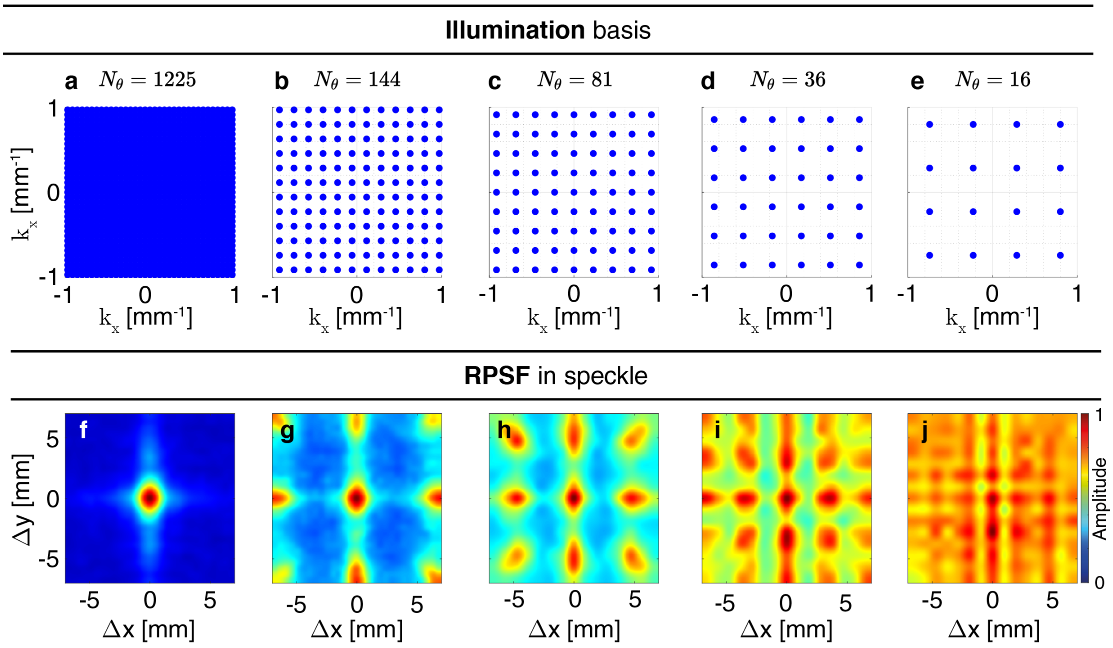

SUPPLEMENTARY FIGURE 8. **Illumination sequence.** (**a-e**) Representation of different plane wave illumination sequence in the **k**−space. (**f-j**) Aliasing effect exhibited by the RPSFs due to incompleteness of illumination sequence displayed in panels a-e, respectively. These RPSFs have been measured in a speckle area of a tissue-mimicking phantom.

illumination basis:

$$\Delta\rho_{\max} \sim \lambda_c/(2\delta\theta) \tag{27}$$

with λ_c the central wavelength and $\delta\theta$ the angular pitch used for the illumination sequence. Thus, to avoid spatial aliasing, the coefficients $R(\boldsymbol{\rho}_{\text{in}}, \boldsymbol{\rho}_{\text{out}}, z)$ associated with a transverse distance $|\boldsymbol{\rho}_{\text{out}} - \boldsymbol{\rho}_{\text{in}}|$ larger than the superior bound $\Delta\rho_{\max}$ should be filtered via a confocal filter.

Equation 27 implies the necessity of recording a high-dimension **R**−matrix for transcranial imaging, as aberrations are particularly important in that configuration (see Fig. 5 of the accompanying paper). The number of independent incident waves should scale as the number of resolution cells over which the RPSF spreads.

XI. DISCRIMINATE MULTIPLE SCATTERING FROM ELECTRONIC NOISE

We consider here the background of the focused reflection matrix for a given point $\mathbf{r}_\mathrm{p}$:

$$B(\boldsymbol{\Delta\rho}, \mathbf{r}_p) = \langle R_{\mathcal{M}}(\boldsymbol{\Delta\rho}, \mathbf{r}_\mathrm{m})\mathcal{D}(\boldsymbol{\Delta\rho})\mathcal{W}(\mathbf{r}_\mathrm{m} - \mathbf{r}_p)\rangle_{\mathbf{r}_\mathrm{m}} \tag{28}$$

where $\mathcal{D}(\Delta\boldsymbol{\rho})$ is a de-scanned window function that eliminates the confocal peak and $\mathcal{W}$ is a spatial average window function around the targeted focal point $\mathbf{r}_\mathrm{p}$.

The background can be decomposed as the sum of a fully symmetric matrix associated to multiple scattering (due to spatial reciprocity) and a fully random matrix associated to the electronic noise as follows:

$$\underbrace{\mathbf{B}}_{\text{Background}} = \underbrace{\mathbf{M}}_{\text{Multiple scattering}} + \underbrace{\mathbf{N}}_{\text{Noise}} \tag{29}$$

Projecting the $\mathbf{B}-$matrix onto its anti-symmetric subspace directly holds the anti-symmetric part of the electronic noise such that:

$$\mathbf{B}^{(\mathrm{A})} = \frac{\mathbf{B} - \mathbf{B}^\top}{2} = \mathbf{N}^{(\mathrm{A})} \tag{30}$$

Assuming equi-repartition of the electronic noise onto its symmetric and anti-symmetric subspace leads to:

$$\|\mathbf{B}^{(\mathrm{A})}\|^2 = \|\mathbf{N}^{(\mathrm{A})}\|^2 = \frac{1}{2}\|\mathbf{N}\|^2 \tag{31}$$

The norm of the background can be expressed as follows:

$$\|\mathbf{B}\|^2 = \|\mathbf{M}\|^2 + \|\mathbf{N}\|^2 + 2\underbrace{\langle \mathbf{M}|\mathbf{N}\rangle}_{\sim 0} \tag{32}$$

Assuming that the scalar product between the electronic noise and the multiple

scattering is zero on average, the multiple scattering rate α_M can be derived by combining equations (31) & (32):

$$\alpha_M = \frac{\|\mathbf{M}\|^2}{\|\mathbf{B}\|^2} = 1 - 2\underbrace{\frac{\|\mathbf{B}^{(\mathrm{A})}\|^2}{\|\mathbf{B}\|^2}}_{\beta} \tag{33}$$

with β the anti-symmetric rate of the $\mathbf{B}-$matrix.

XII. NOTATION AND SYMBOLS

Symbol	Meaning
$\mathbf{R}$	Reflection matrix
$\mathbf{H}$	Point spread function matrix
$RPSF$	Reflection point spread function
$\mathbf{G}$	Propagation matrix
$\mathbf{D}$	Distortion matrix
$\mathbf{C}$	Correlation matrix
$\delta\mathbf{C}$	Perturbation term of $\mathbf{C}$
$\mathbf{T}$ and $\hat{\mathbf{T}}$	Transmission matrix and its estimator
$\|\delta T\|^2$	Bias intensity of $\mathbf{T}-$matrix estimator
$P_{\mathrm{in/out}}$	Scalar product between $\hat{\mathbf{T}}_{\mathrm{in}}$ and $\hat{\mathbf{T}}_{\mathrm{out}}$
$\mathbf{i}$	Illumination basis
$\mathbf{c}$	Correction basis
$\mathbf{u}$	Transducer basis
$\mathbf{k}$	Fourier basis
$\boldsymbol{\theta}$	Plane wave basis
l_c	Confocal filter size
ITR	Iterative Time Reversal
IPR	Iterative Phase Reversal
$\mathbf{W}^{(n)}$	Wave-front of the ITR process at iteration n
$\mathbf{r}_m$	Common midpoint
$\mathbf{r}_p$	Central point of a patch
$\boldsymbol{\Delta\rho} = \boldsymbol{\rho}_{\mathrm{out}} - \boldsymbol{\rho}_{\mathrm{in}}$	Distance input/output focusing points
$\mathcal{D}(\boldsymbol{\Delta\rho})$	De-scanned window function

SUPPLEMENTARY TABLE I. **List of symbols for matrix imaging.**

Basis	Symbol	Adapted for
Acquisition basis	$\mathbf{R_{iu}}(t) = [R(\mathbf{i}_{\text{in}}, \mathbf{u}_{\text{out}}, t)]$	Data recording
Focused basis<br>Common midpoint	$\mathbf{R}_{\boldsymbol{\rho\rho}}(z) = [R(\boldsymbol{\rho}_{\text{in}}, \boldsymbol{\rho}_{\text{out}}, z)]$<br>$\mathbf{R}_{\mathcal{M}}(z) = [R(\boldsymbol{\Delta\rho}, \boldsymbol{\rho}_{\text{m}}, z)]$	Focusing quality and multiple scattering quantification[2]
Dual basis (input)	$\mathbf{R_{cr}} = [R(\mathbf{c}_{\text{in}}, \mathbf{r}_{\text{out}})]$<br>$\mathbf{D_{cr}} = [D(\mathbf{c}_{\text{in}}, \mathbf{r}_{\text{out}})]$<br>$\mathbf{C}_{\text{in}} = [C(\mathbf{c}_{\text{in}}, \mathbf{c}'_{\text{in}})]$<br>$\hat{\mathbf{T}}_{\text{in}} = [\hat{T}(\mathbf{c}_{\text{in}}, \mathbf{r}_{\text{p}})]$	Local aberration compensation[7]
Dual basis (output)	$\mathbf{R_{rc}} = [R(\mathbf{r}_{\text{in}}, \mathbf{c}_{\text{out}})]$<br>$\mathbf{D_{rc}} = [D(\mathbf{r}_{\text{in}}, \mathbf{c}_{\text{out}})]$<br>$\mathbf{C}_{\text{out}} = [C(\mathbf{c}_{\text{out}}, \mathbf{c}'_{\text{out}})]$<br>$\hat{\mathbf{T}}_{\text{out}} = [\hat{T}(\mathbf{r}_{\text{p}}, \mathbf{c}_{\text{out}})]$	

SUPPLEMENTARY TABLE II. **Matrix notations.**

Symbol	Meaning
$\times$	Matrix product
$\circ$	Hadamard product
$\circledast$	Convolution product
$\dagger$	Transpose conjugate of a matrix
$\top$	Matrix transpose
$\hat{}$	Estimator of a physical quantity
SVD	Singular Value Decomposition
$\mathbf{U}^{(i)}$	i^{th} right singular vector of a matrix
$\mathbf{V}^{(i)}$	i^{th} left singular vector of a matrix
σ_i	i^{th} singular value of a matrix
$\langle \cdots \rangle$	Ensemble average

SUPPLEMENTARY TABLE III. **Mathematical symbols.**

Symbol	Meaning
$\mathcal{I}$	Image ⇔ Estimation of the reflectivity
$\mathbf{r} = (x, y, z)$	Focal point
$\boldsymbol{\rho} = (x, y)$	Transverse coordinate
λ_c	Wavelength at the central frequency
f_s	Sampling frequency
f_c	Central frequency
c_0	Speed-of-sound hypothesis
$\mathbf{u} = (u_x, u_y, 0)$	Transducer position
$\delta\rho_0$	Transverse ideal resolution
t	Time
τ	Time-of-flight
$\Delta\tau$	Time-delay
γ	Medium reflectivity
$\boldsymbol{\theta} = [\theta_x, \theta_y]$	Plane wave
$\mathbf{k} = [k_x, k_y]$	Fourier basis
β	Anti-symmetric rate of a matrix
θ_{max}	Directivity of transducers
$\delta\theta$	Plane wave sampling
$\boldsymbol{\Delta}\mathbf{u} = (\Delta u_x, \Delta u_y)$	Probe dimension
$\mathcal{C}$	Coherence factor
$\mathcal{A}_{(-3\text{dB})}$	Area above 1/2 on RPSF amplitude
$\delta\rho_{(-3\text{dB})}$	Experimental RPSF resolution
$\delta\rho_0$	Diffraction-limited resolution
$\mathcal{F}$	RPSF contrast
α_{S}	RPSF single scattering rate
α_{M}	RPSF multiple scattering rate
α_{N}	RPSF electronic noise rate
α_{B}	RPSF background rate
$\mathcal{W}$	Spatial average window function
$N_{\mathcal{W}}$	Number of resolution cells in $\mathcal{W}$
$\mathbf{w} = (w_\rho, w_z) = (\{w_x, w_y\}, w_z)$	Dimension of $\mathcal{W}$
A	Apodization term of synthetic aperture

SUPPLEMENTARY TABLE IV. **List of general symbols.**

SUPPLEMENTARY REFERENCES

[1] W. Lambert, L. A. Cobus, T. Frappart, M. Fink, and A. Aubry, Distortion matrix approach for ultrasound imaging of random scattering media, Proc. Nat. Acad. Sci. USA **117**, 14645 (2020).

[2] W. Lambert, J. Robin, L. A. Cobus, M. Fink, and A. Aubry, Ultrasound matrix imaging – Part I: The focused reflection matrix, the F-factor and the role of multiple scattering, IEEE Trans. Med. Imag. **41**, 3907 (2022).

[3] J.-L. Robert and M. Fink, Green's function estimation in speckle using the decomposition of the time reversal operator: Application to aberration correction in medical imaging, J. Acoust. Soc. Am. **123**, 866 (2008).

[4] S. Kang, P. Kang, S. Jeong, Y. Kwon, T. D. Yang, J. H. Hong, M. Kim, K. Song, J. H. Park, J. H. Lee, M. J. Kim, K. H. Kim, and W. Choi, High-resolution adaptive optical imaging within thick scattering media using closed-loop accumulation of single scattering, Nat. Commun. **8**, 2157 (2017).

[5] R. Mallart and M. Fink, Adaptive focusing in scattering media through sound-speed inhomogeneities: The van Cittert Zernike approach and focusing criterion, J. Acoust. Soc. Am. **96**, 3721 (1994).

[6] S. Silverstein, Ultrasound scattering model: 2-d cross-correlation and focusing criteria-theory, simulations, and experiments, IEEE Trans. Ultrason. Ferroelectr. Freq. Control **48**, 1023 (2001).

[7] W. Lambert, L. A. Cobus, J. Robin, M. Fink, and A. Aubry, Ultrasound matrix imaging – Part II: The distortion matrix for aberration correction over multiple isoplanatic patches, IEEE Trans. Med. Imag. **41**, 3921 (2022).
